# Supplementary material for: The conformational changes coupling ATP hydrolysis and translocation in a bacterial DnaB helicase
Source: Nat Commun. 2019 Jan 3;10:31. doi: 10.1038/s41467-018-07968-3 (PMC6318325; doi:10.1038/s41467-018-07968-3)
Supplement: Supplementary file 1 — Supporting Information [file 41467_2018_7968_MOESM1_ESM.pdf]

**Supplementary material for:**

# The conformational changes coupling ATP hydrolysis and translocation in a bacterial DnaB helicase

Wiegand et al.

## Supplementary Notes

*Stoichiometry of ATP-analogue used for the BstDnaB:DNA complex*[1]

17 µM $(DnaB)_6$ relates to 0.102 mM DnaB monomers. 10 mM $MgCl_2$ (~100-fold excess compared to one DnaB monomer), 2 mM GDP (~20-fold excess) and 0.5 mM $AlF_3$/5 mM NaF (resulting in an ~5-fold excess of $AlF_4^-$ which is the limiting concentration in this case) were added to DnaB.

*Structural models of HpDnaB used for visualizing CSPs*

In this work, two structural models of *Hp*DnaB were used. In Figures 2 to 4, the model of a DnaB hexamer based on the full-length *Hp*DnaB X-ray structure (pdb accession code 4ZC0) was employed. The crystals were obtained in the presence of ADP:$Mg^{2+}$, but, no electron density for ADP:$Mg^{2+}$ was observed at the given resolution[2]. In Figure 5, a homology model of the *Hp*DnaB:ADP:$Mg^{2+}$ complex based on the crystal structure of *Aa*DnaB:ADP:$Mg^{2+}$ (pdb accession code 4NMN)[3] was used.

## Supplementary Figures

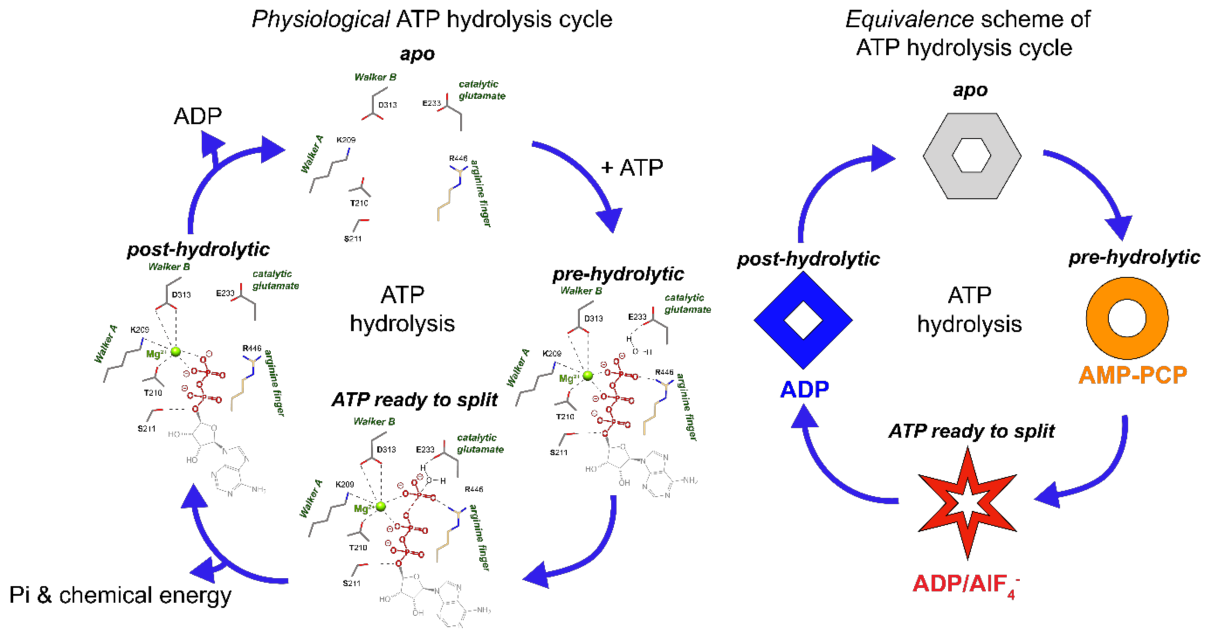


**Supplementary Figure 1:** *ATP-analogues allow to study different conformations of the DnaB helicase mimicking the ones adopted during ATP hydrolysis.* Schematic representation of ATP-hydrolysis by the *Hp*DnaB helicase (left: physiological cycle, right: equivalence cycle used in this work). The amino acids of *Hp*DnaB involved in ATP-binding and hydrolysis are shown together with the corresponding state of ATP in the left scheme.

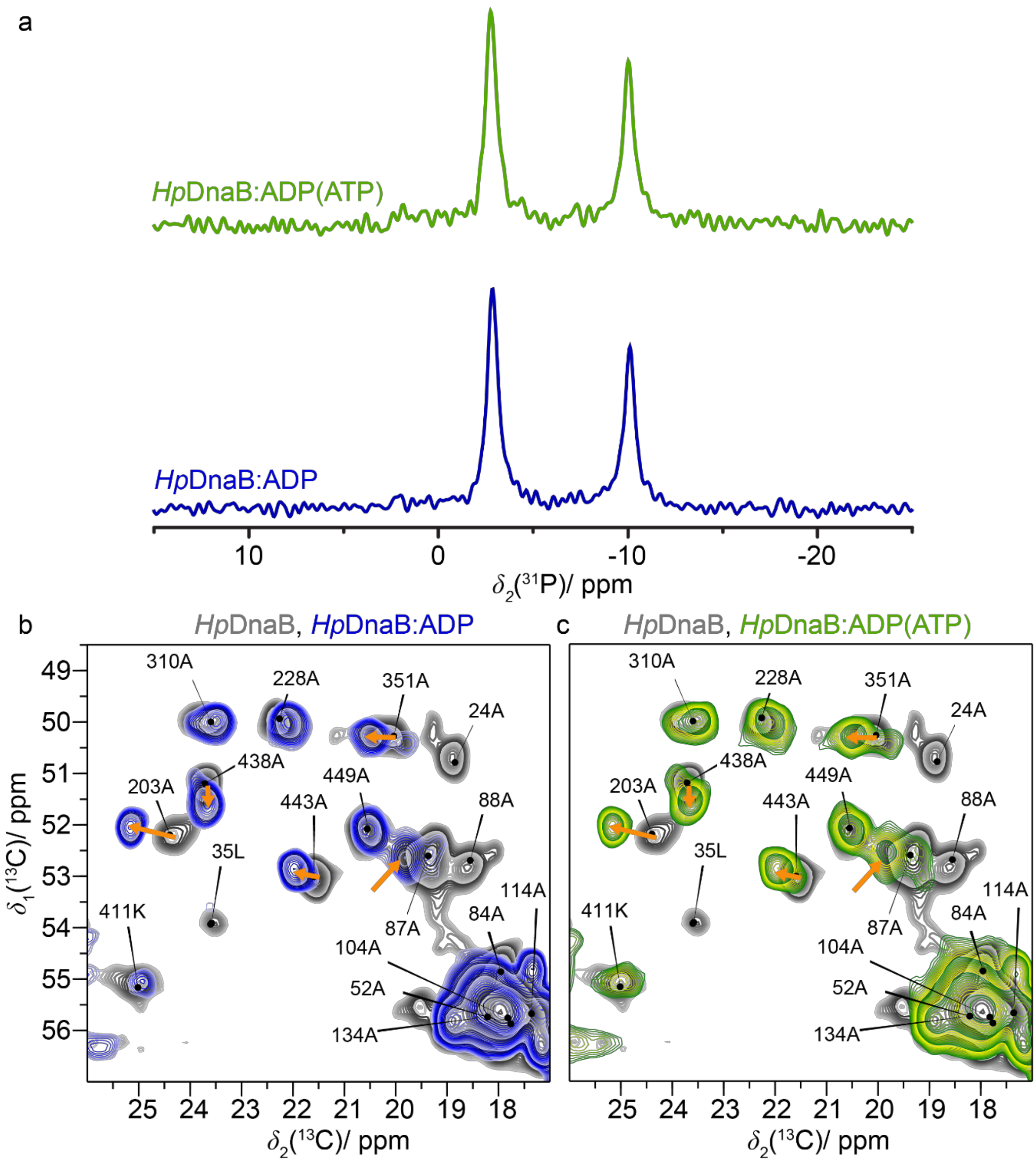


**Supplementary Figure 2**: *ATP-hydrolysis occurs on the time scale of sample preparation.* **a** Comparison of $^{31}$P,$^{1}$H CP-MAS spectra of DnaB:ADP (blue) and DnaB:ADP(ATP) (green) for which ATP was added to the protein instead of ADP. For the spectrum in blue only ADP was added to the protein, for the spectrum shown in green only ATP. After 2 h incubation, the protein was sedimented for 16 h in the NMR rotors. The experiment was recorded ~24 h after the incubation step. Note that proteins sedimented in an NMR rotor are hydrated to roughly 50 %. Therefore, ATP hydrolysis still continues in the NMR rotor. **b** and **c** Extracts of 20 ms $^{13}$C-$^{13}$C DARR spectra of *apo* DnaB (grey), DnaB:ADP (blue) and DnaB:ADP(ATP) (green). The spectra obtained upon ADP and ATP addition are identical indicating that under both conditions, only the ADP-bound state is obtained. Orange arrows indicate $^{13}$C CSPs. The *apo* spectrum is taken from reference [4].

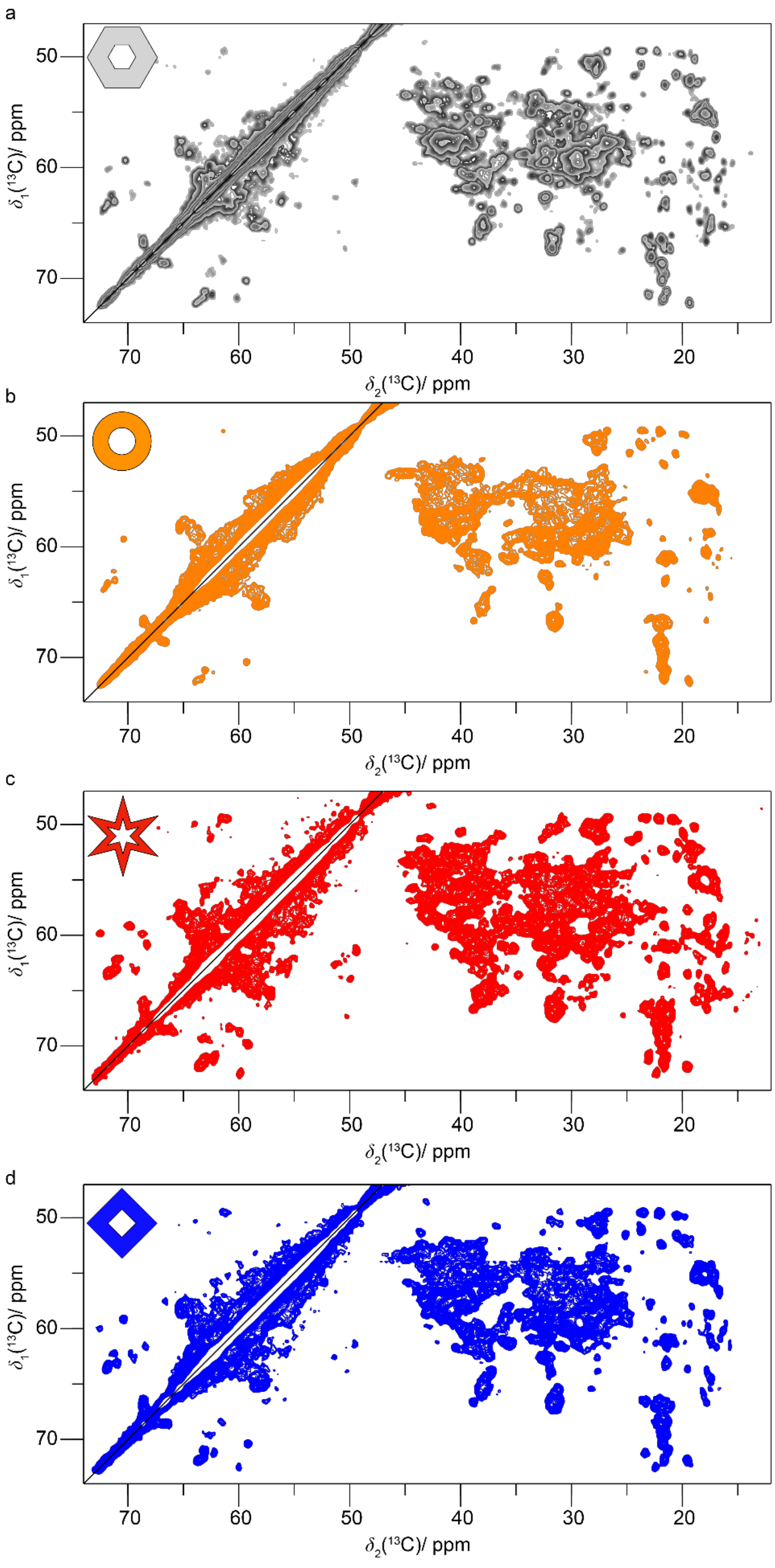


**Supplementary Figure 3:** *Solid-state NMR allows to distinguish different states of the ATP hydrolysis equivalence scheme.* Aliphatic regions of $^{13}C$-$^{13}C$ 20 ms DARR spectra for the states of the artificial ATP hydrolysis cycle (**a**: *apo HpDnaB*, **b**: DnaB:AMP-PCP, **c**: DnaB:ADP:$AlF_4^-$ and **d**: DnaB:ADP). The *apo* spectrum is taken from reference [4].

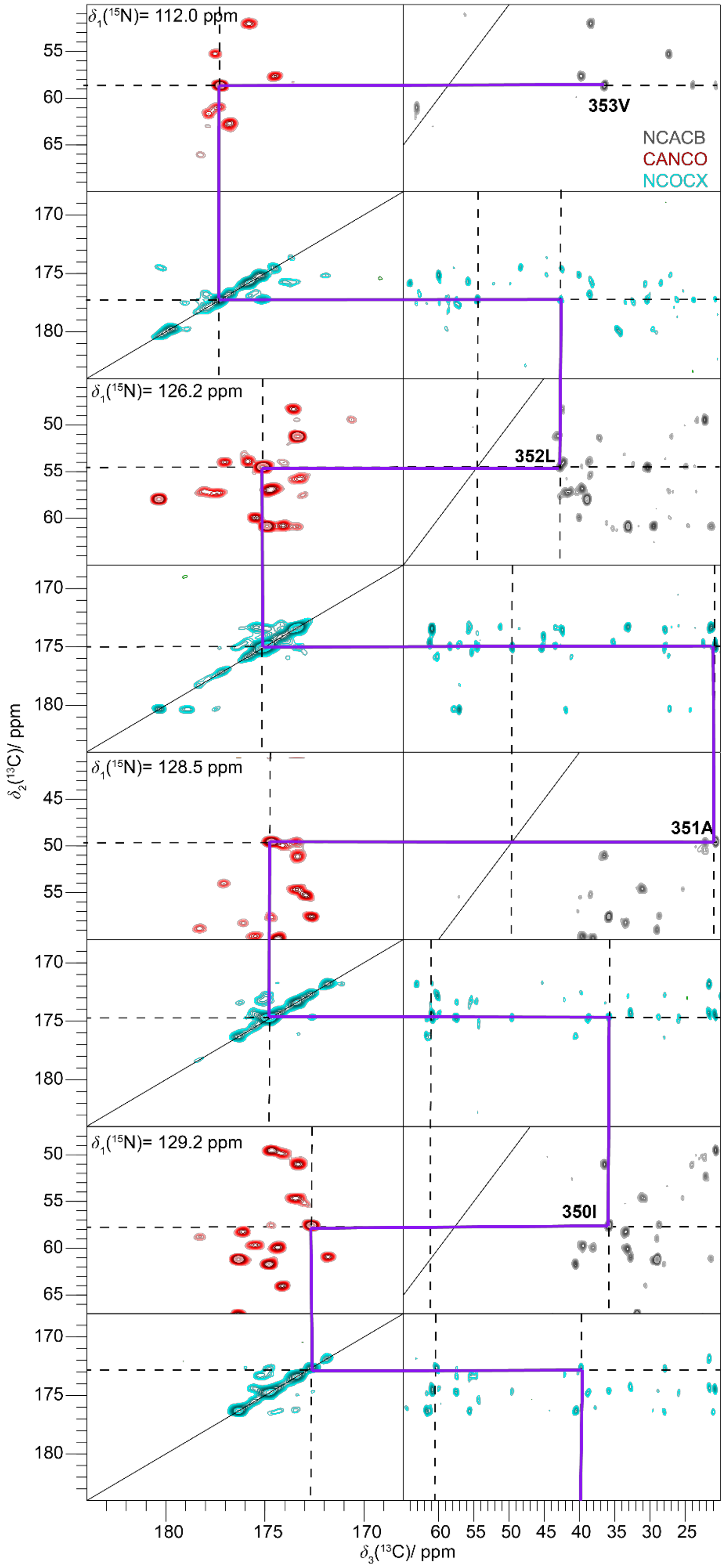


**Supplementary Figure 4**: *Sequential backbone walk for resonance assignment using conventional 3D experiments.* Representative example for a sequential backbone walk for the

DnaB:ADP:$AlF_4^-$:ssDNA complex showing 2D planes of 3D NCACB, CANCO and NCOCX spectra.

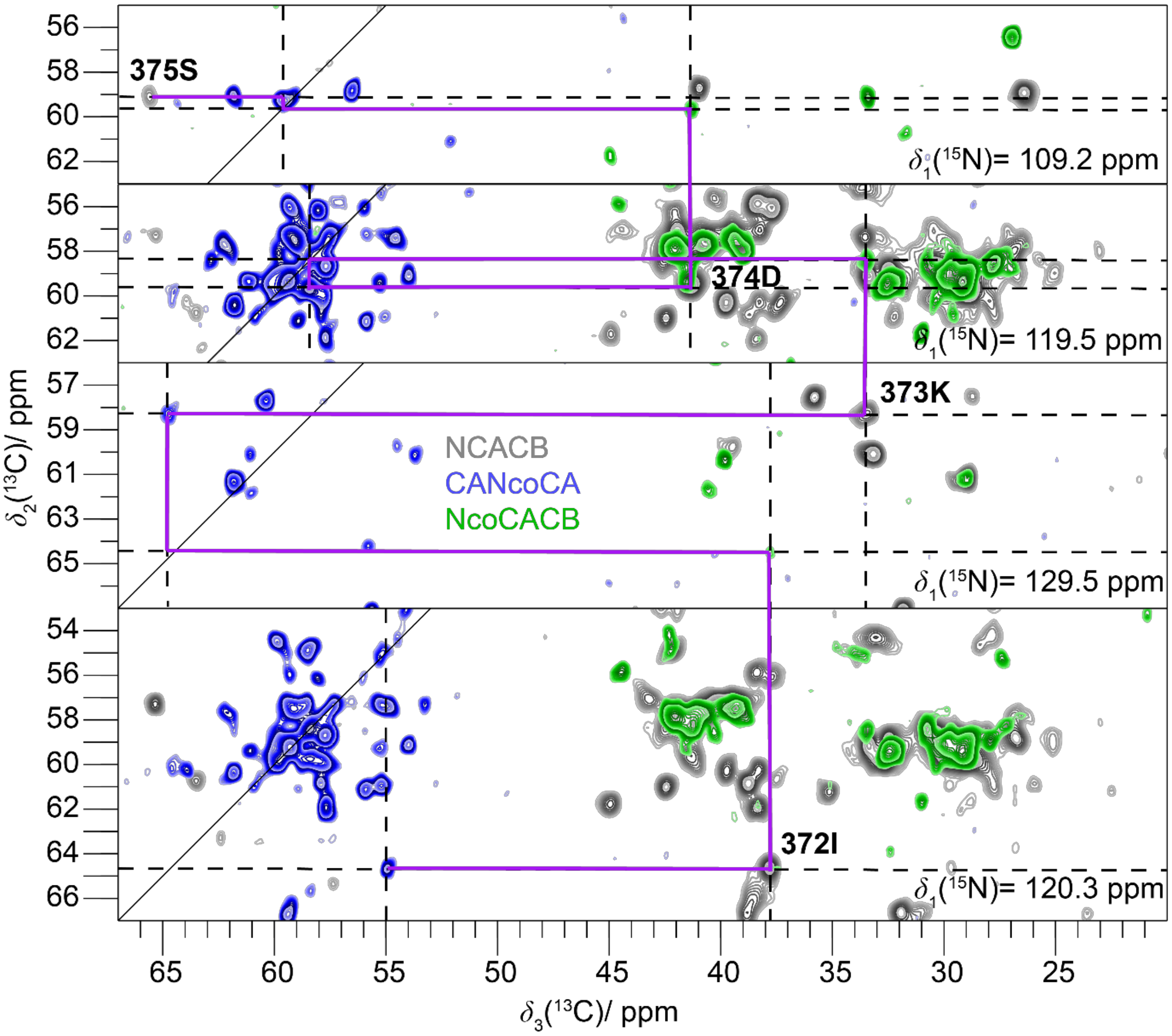


**Supplementary Figure 5**: *Sequential backbone walk for resonance assignment using only relayed 3D experiments.* Representative example for a sequential backbone walk for the DnaB:ADP:$AlF_4^-$:ssDNA complex showing 2D planes of 3D NCACB, CANcoCA and NcoCACB spectra. These spectra complement those shown in the previous Figure.

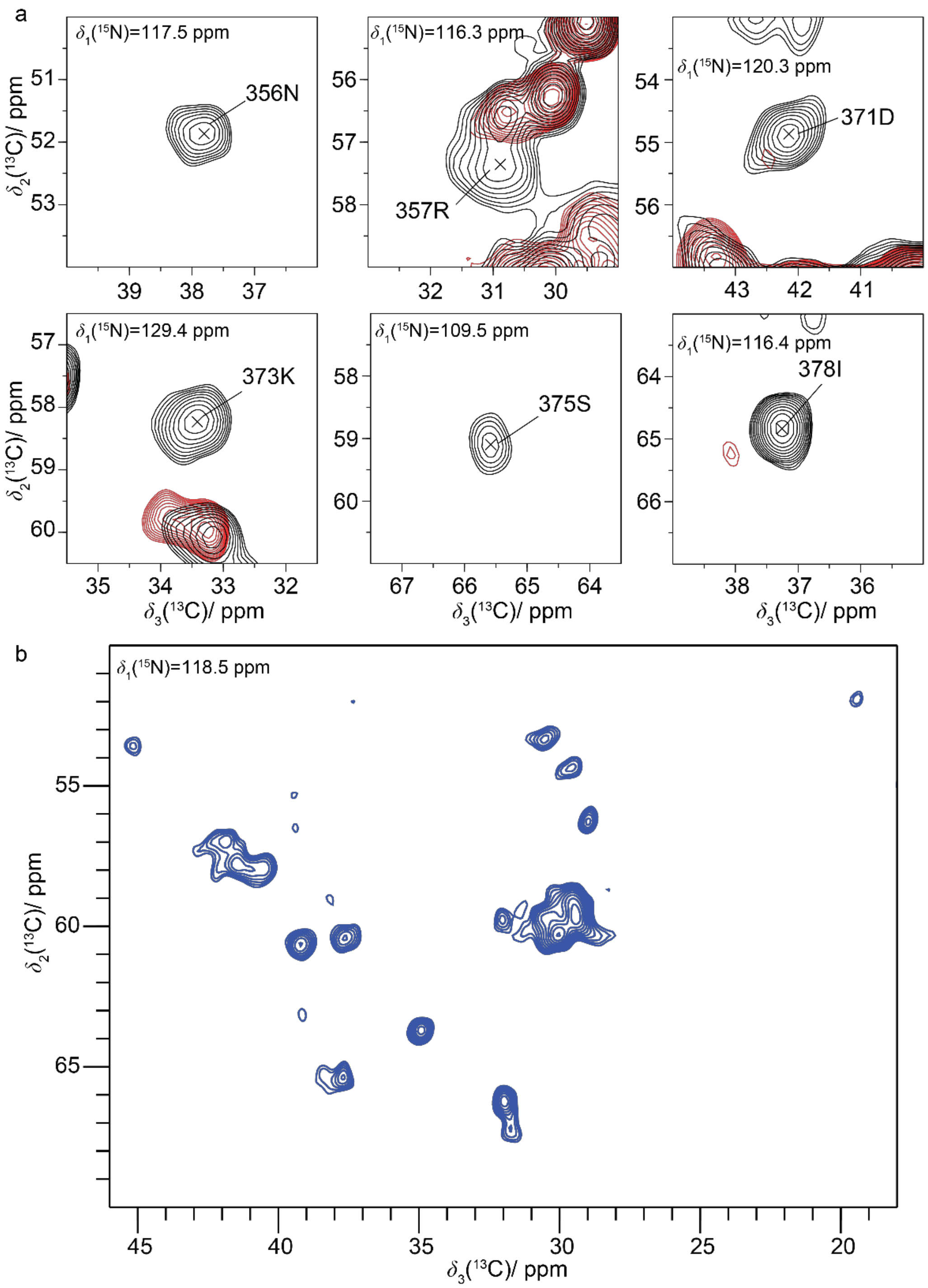


**Supplementary Figure 6**: *3D spectra reduce spectral overlap.* **a** Representative 2D planes of 3D NCACB spectra of DnaB:ADP:AlF$_4^-$ (red) and DnaB:ADP:AlF$_4^-$:ssDNA (grey) illustrating the appearance of peaks upon DNA binding. **b** Representative plane of a 3D NCACB spectrum of apo DnaB in which only the NTD is 13C/15N labelled. For more details see reference [5].

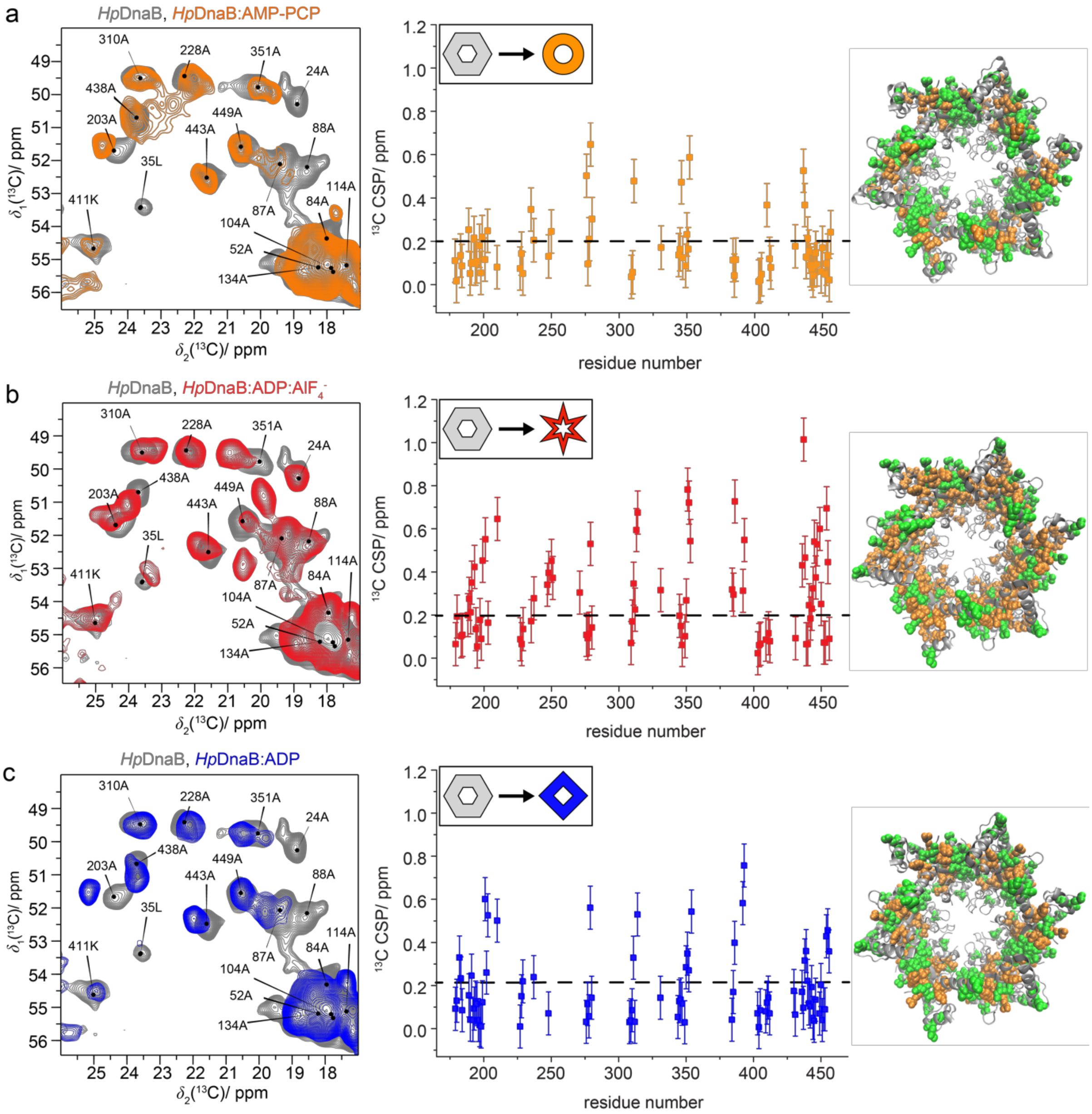


**Supplementary Figure 7:** *Conformational differences in DnaB upon ATP-analogue binding.* Spectral fingerprints of $^{13}$C-$^{13}$C 20 ms DARR correlation spectra (first column) and $^{13}$C backbone CSPs (second column) for the studied ATP-analogue bound states of DnaB employing **a** AMP-PCP, **b** ADP:$AlF_4^-$, and **c** ADP. The assignments shown in the spectra are taken from the *apo* DnaB protein. The error bars (identical for all residues) are estimated to 0.1 ppm from the differences in the experimental spectra. The last column shows the $^{13}$C backbone CSPs plotted on the DnaB low-resolution structure (green: CSP < 0.2 ppm, orange: CSP ≥ 0.2 ppm). All source data for the chemical shift values of columns 2 and 3 are listed in the Supplementary Tables 2.

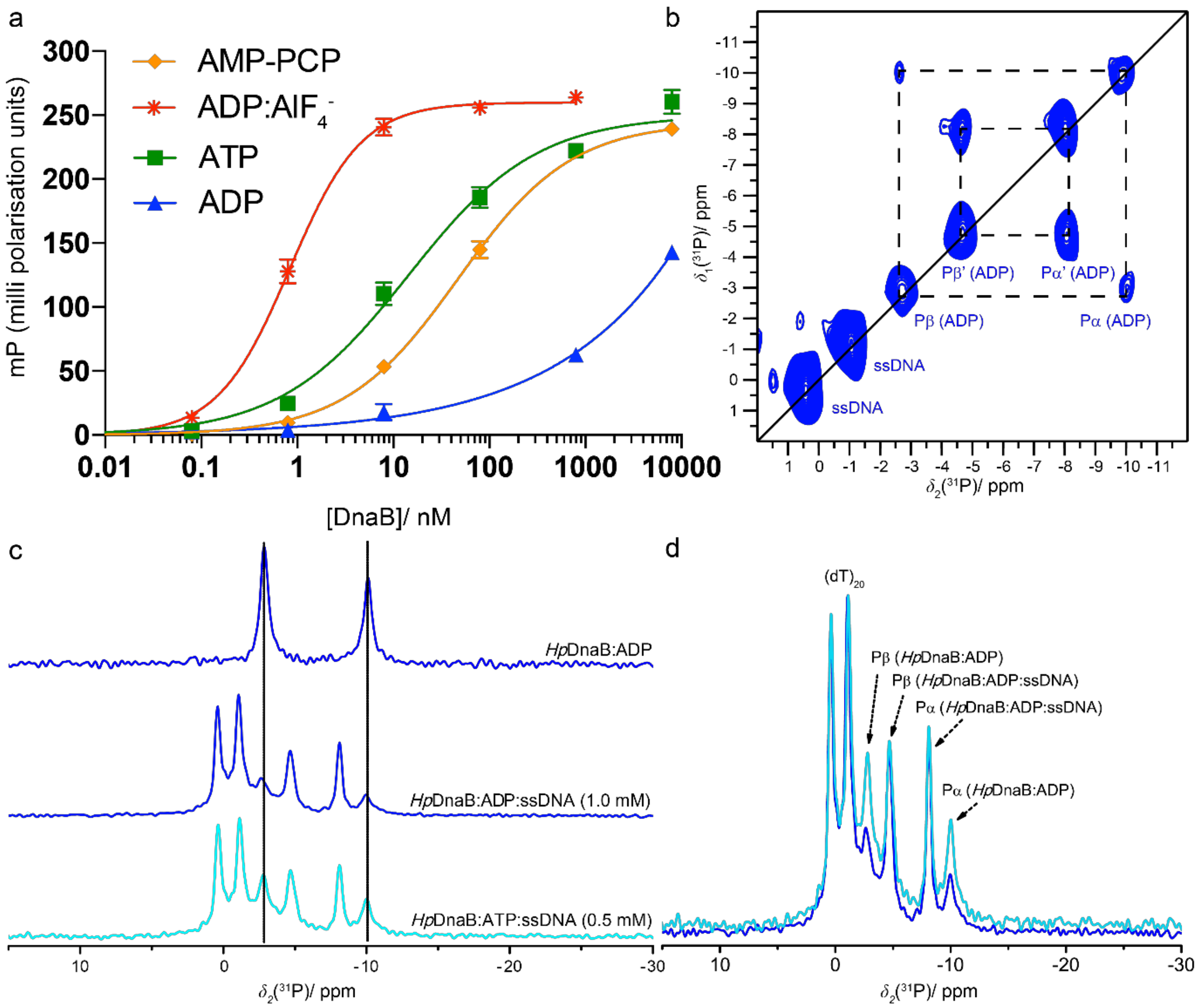


**Supplementary Figure 8**: **a** *$ADP:AlF_4^-$ shows highest binding affinities towards ssDNA*. Fluorescence anisotropy measurements of *Hp*DnaB binding to a 5'-FAM labeled 20mer ssDNA ($dT_{20}$) in the presence of ATP-analogues. $ADP:AlF_4^-$ as the best binder is followed by ATP. ADP binds ssDNA with much lower affinity. For the details of the fits see Supplementary Table 7. Note that the fluorescence anisotropy measurements were performed on different samples (another batch) than the solid-state NMR studies. **b-d** *$^{31}P$ NMR reveals ssDNA- and ADP-binding to the helicase. The $^{31}P$ spectrum is clearly different with and without ssDNA bound.* **b** $^{31}P$-$^{31}P$ correlation spectrum (employing 150 ms DARR mixing) of the DnaB:ATP:ssDNA complex (1 mM ssDNA concentration) highlighting two ADP spin-systems: one belongs to DnaB:ADP and the other one to the DnaB:ADP:ssDNA complex. **c** $^{31}P$,$^{1}H$ CPMAS spectra of DnaB:ADP:ssDNA samples with different ssDNA concentrations (cyan: 0.5 mM and blue: 1 mM) which are compared with the spectrum of DnaB:ADP in the absence of ssDNA. The data clearly show that ADP and ssDNA are bound to the helicase and that not all DnaB:ADP complexes have reacted with ssDNA (the amount of ssDNA-bound species increases for higher ssDNA concentrations). Two resonances of ssDNA in a 1:1 ratio are observed in agreement with the expectation that two nucleotides bind per DnaB monomer. **d** *The $^{31}P$ resonances belonging to the DnaB:ADP:ssDNA sample are unambiguously identified by concentration dependent experiments: the relative intensity ratio of ssDNA and ADP $^{31}P$ resonances is conserved in these studies.* Overlay of $^{31}P$,$^{1}H$ CPMAS spectra of the DnaB:ADP:DNA samples

with ssDNA concentrations of 0.5 mM (cyan) and 1 mM (blue). The sample at highest ssDNA concentration still contains a minor amount of *Hp*DnaB:ADP.

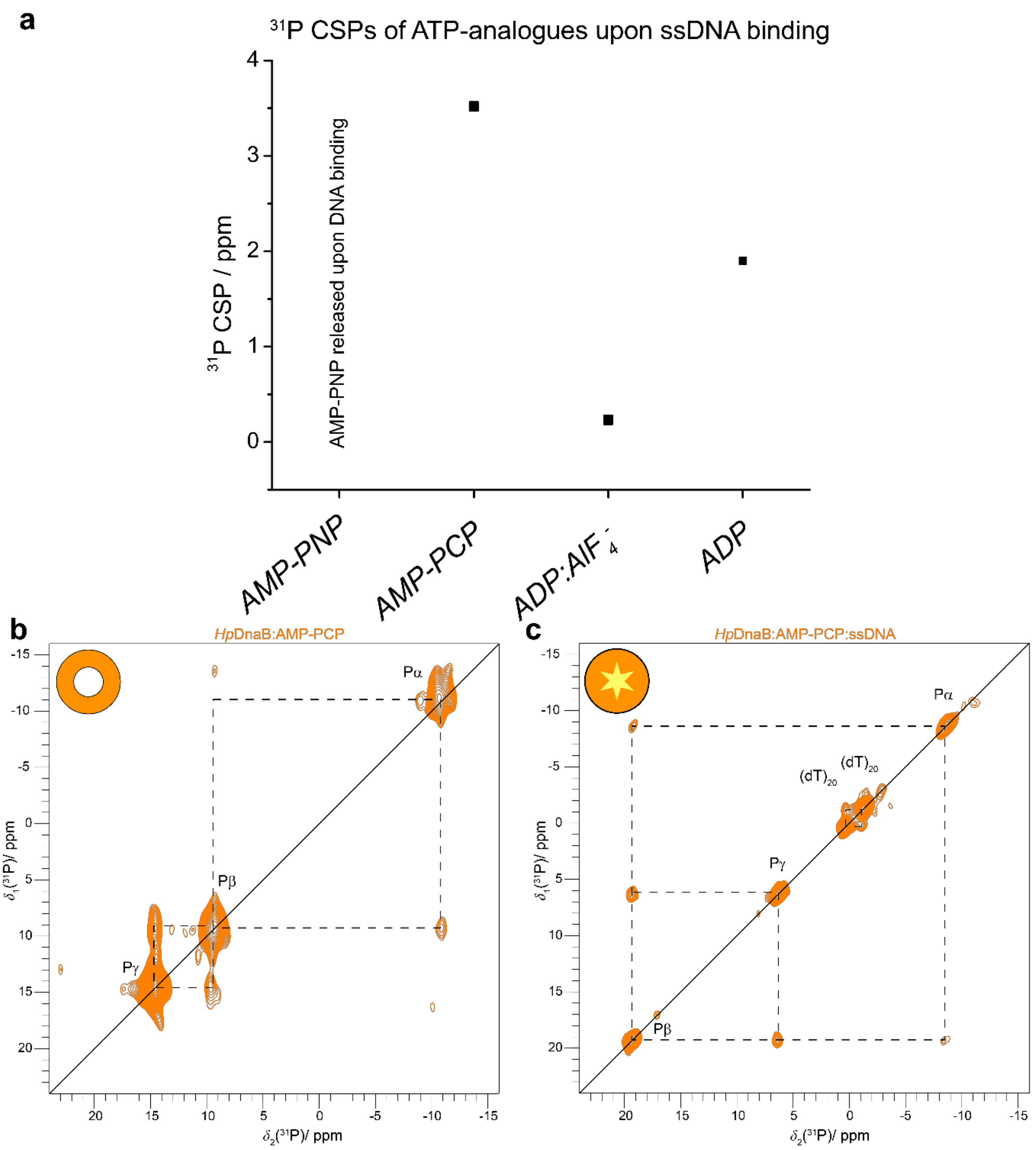


**Supplementary Figure 9**: **a** *$^{31}$P CSPs (DnaB:ATP-analogue:ssDNA vs. DnaB:ATP-analogue) indicate structural preorganization of the motor domain by ADP:$AlF_4^-$.* The $^{31}$P CSPs indicate structural changes of the bound ATP-analogue upon ssDNA-binding. In case of AMP-PCP and ADP sizeable CSPs are observed, while in case of AMP-PNP we have observed the hydrolysis to AMP-PNP in the presence of ssDNA and a subsequent release of the hydrolysed product[6]. For ADP:$AlF_4^-$ nearly no changes were detected. **b** *$^{31}$P-$^{31}$P correlation spectrum (employing 200 ms DARR mixing)of DnaB:AMP-PCP and* **c** *$^{31}$P-$^{31}$P correlation spectrum (employing 150 ms DARR mixing) of DnaB:AMP-PCP:ssDNA indicate different binding conformations of AMP-PCP in the absence and presence of ssDNA.* Note, that in **c** also correlations between the two different DNA nucleotides are detected in accordance with the interpretation that they represent two distinct DNA nucleotides bound to one DnaB monomer.

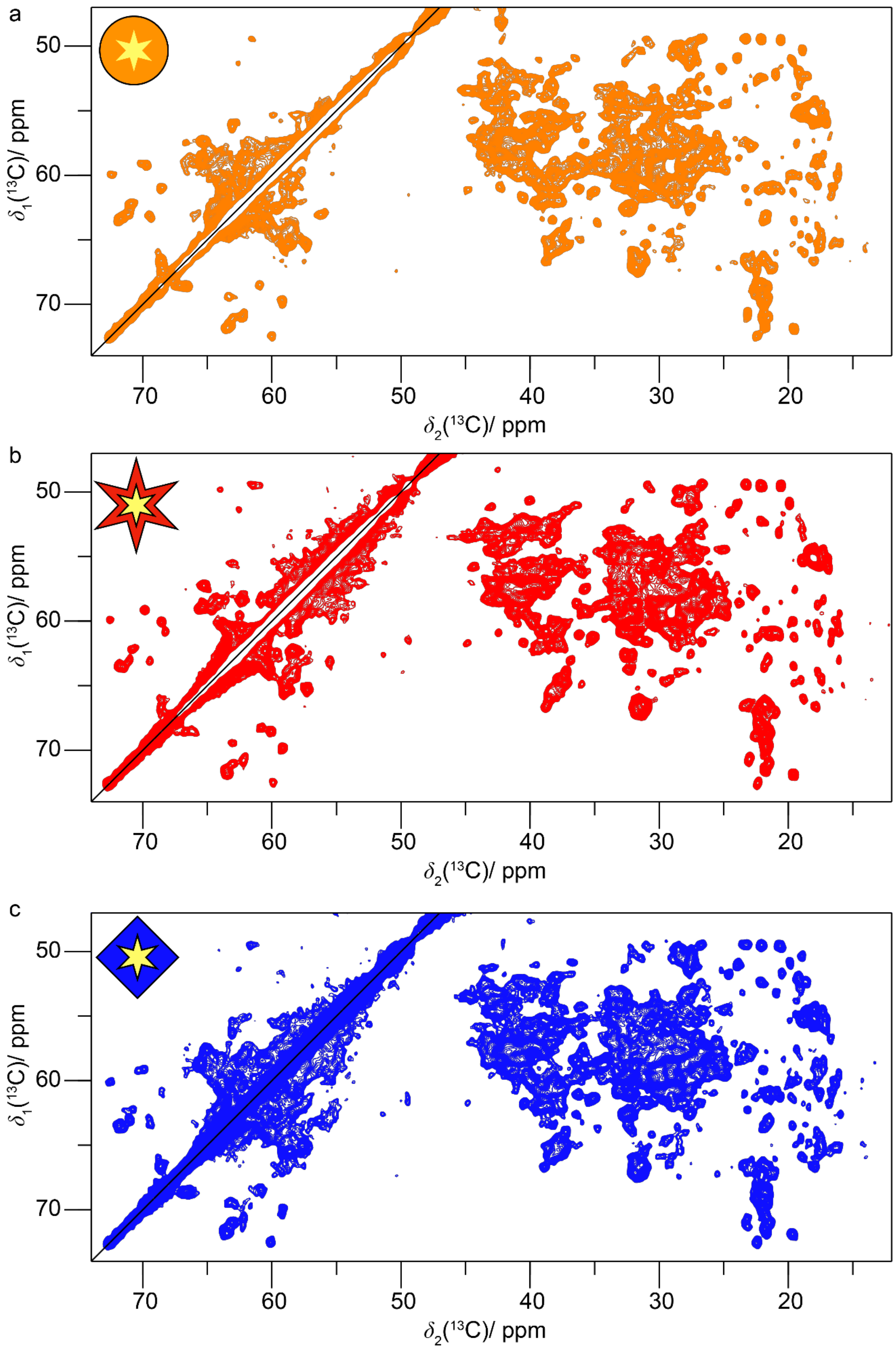


**Supplementary Figure 10**: *Solid-state NMR allows to distinguish different states of DNA translocation.* Aliphatic regions of $^{13}C$-$^{13}C$ 20 ms DARR spectra for the DNA-bound states during DNA translocation (**a** AMP-PCP, **b** ADP:$AlF_4^-$, **c** ADP).

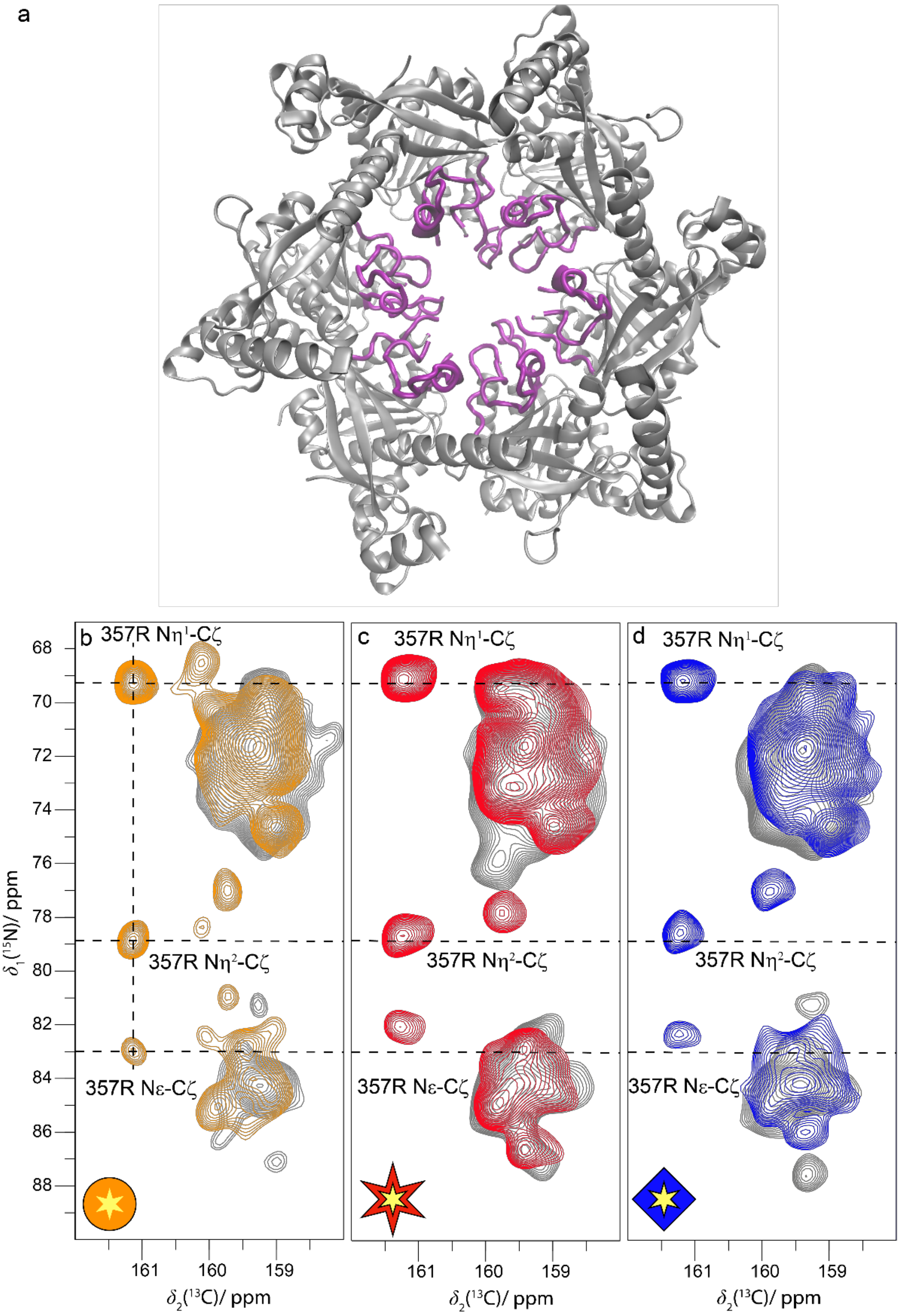


**Supplementary Figure 11**: **a** *DNA binding loops point to the inner channel of the helicase.* Potential DNA binding loops putatively identified (residues 316-330 and 355-383, shown in purple) and highlighted on the structural model based on the low-resolution *Hp*DnaB crystal structure (pdb accession code 4ZC0). **b** *357R is involved in DNA binding and becomes rigid upon ssDNA binding*. Comparison of $^{15}N$,$^{13}C$ correlation spectra for the arginine sidechain for the DNA-bound states using **b** AMP-PCP, **c** ADP:$AlF_4^-$ and **d** ADP. The corresponding spectrum in the absence of ssDNA is shown in grey.

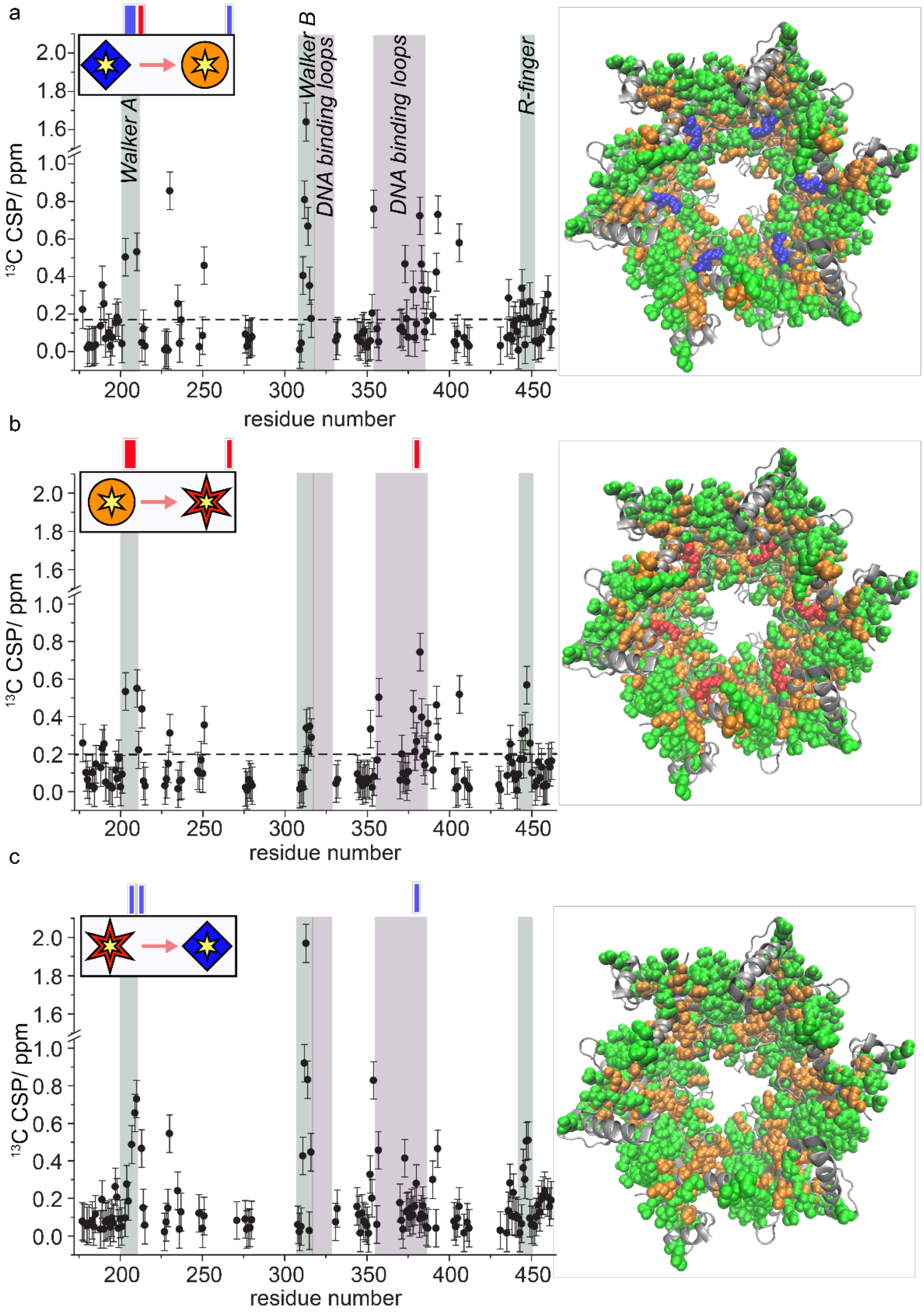


**Supplementary Figure 12:** *NMR allows to follow changes during DNA translocation site-specifically.* $^{13}$C Cα-Cβ CSPs during DNA translocation. Residues newly appearing or disappearing are marked by red or blue spheres, respectively, on the structure and their residue numbers are indicated to guide the eyes by red or light blue bars on top of the CSP plots (see also Supplementary Table 5). The error bars (identical for all residues) are estimated to 0.1 ppm from the differences in the experimental spectra. The second column shows a plot of the $^{13}$C CSPs on a *Hp*DnaB structural model (residues 174-488 are shown, pdb accession code 4ZC0, no electron density for ADP:$Mg^{2+}$ observed[2]) (green: CSP < 0.2 ppm, orange: CSP ≥ 0.2 ppm, red: newly appearing in the spectrum upon DNA binding). All source data for the chemical shift values are listed in the Supplementary Tables 2.

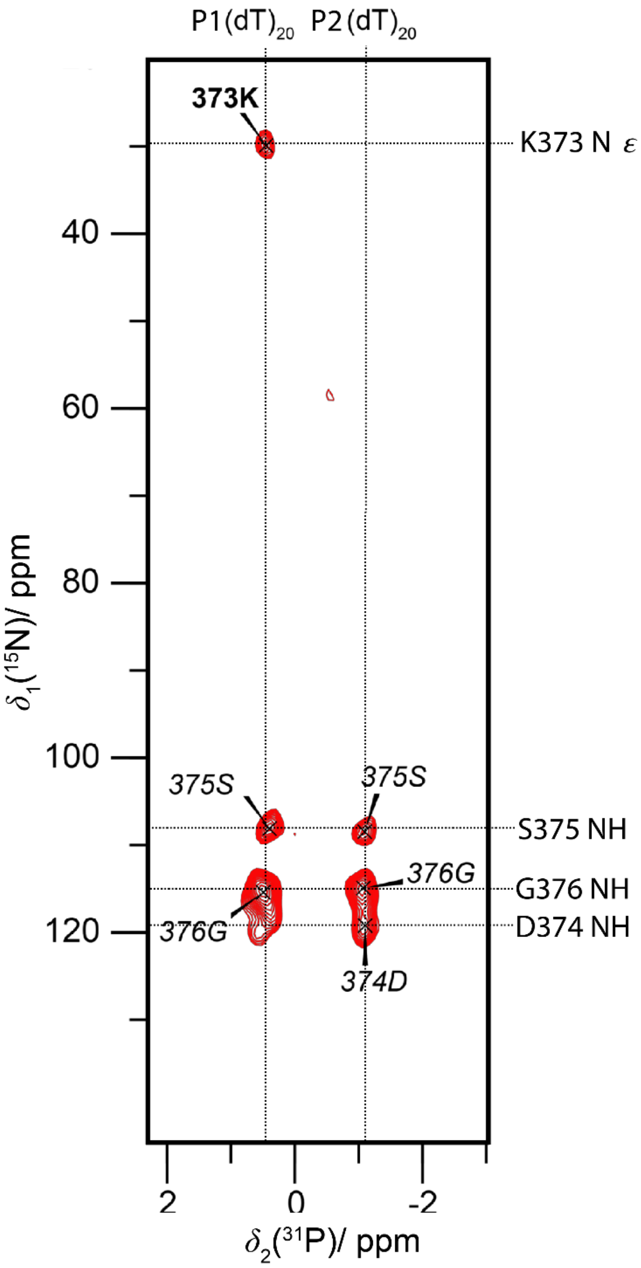


**Supplementary Figure 13:** *Protein-DNA contacts probed by NHHP correlation experiments.* $^{15}N$,$^{31}P$ NHHP correlation spectrum recorded on DnaB:ADP:$AlF_4^-$:DNA. Residues highlighted in italic represent ambiguous assignments, the bold one is unambiguous. One of the DNA phosphate groups is thus in close contact to the sidechain nitrogen atom of 373K. The polarization evolves during a first period of time in the two-dimensional experiment on the nitrogen spins, after cross polarization from the protons. As K373 N$\varepsilon$ has a unique $^{15}N$ chemical shift, determined by sequential assignments using 3D spectroscopy, it can be unambiguously identified. Even if the chemical shifts of the amide nitrogen spins of the neighbouring residues D374, S375 and G376 are not unique, the signals are fully consistent with an assignment to these amide nitrogens adding further evidence. After the transfer to the phosphorous spins via the protons exploiting proton spin diffusion, the polarization is detected on the phosphorous spins, and in the second dimension, one can read the $^{31}P$ frequency, of two different phosphate spins from DNA, of which only one is in close proximity (ca. 3-4 Å) to K373 N$\varepsilon$, since only the $^{31}P$ around 0.5 ppm shows a cross signal with it. The other $^{31}P$ site, at -1 ppm shows a correlation to D374, S375 and G376, and is thus not located far from the first one.

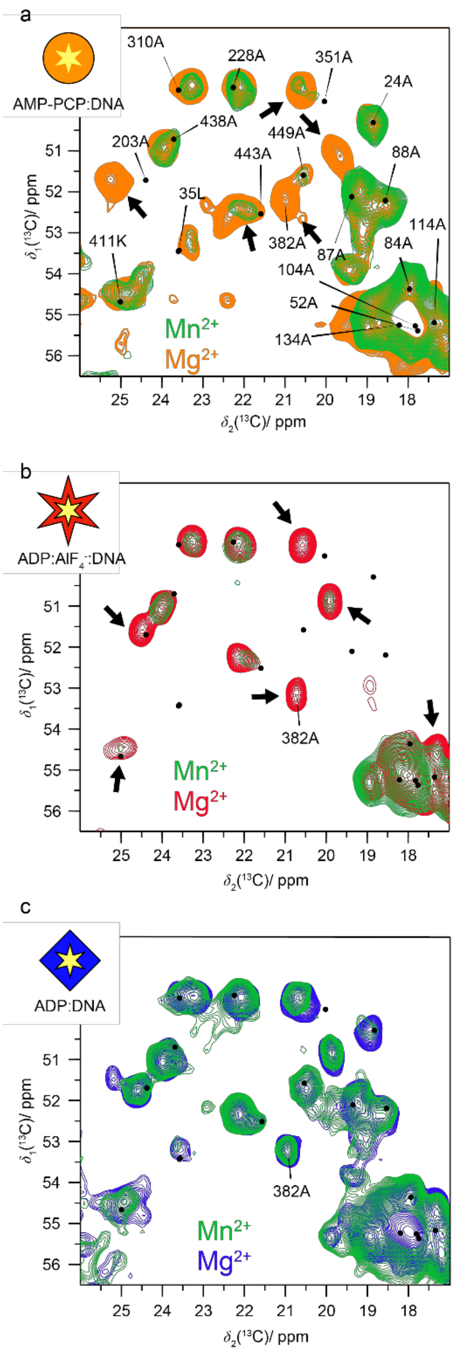


**Supplementary Figure 14**: *Metal ion cofactor is released in the last step of DNA translocation.* Comparison of 2D $^{13}C$-$^{13}C$ 20 ms DARR alanine fingerprints of **a** AMP-PCP:DNA (orange with $Mg^{2+}$, green with $Mn^{2+}$), **b** ADP:$AlF_4^-$:DNA (red with $Mg^{2+}$, green with $Mn^{2+}$), **c** ADP:DNA (blue with $Mg^{2+}$, green with $Mn^{2+}$). Many peaks are disappearing in the case of the paramagnetic samples in **a** and **b** due to strong PRE effects indicating the binding of the metal to the nucleotide and thus to the NBDs. In case of **c** such an effect is not observed pointing to the release of the metal ion in this case. The assignments are those of the full-length protein, except 382A which is taken from the DNA-bound complexes.

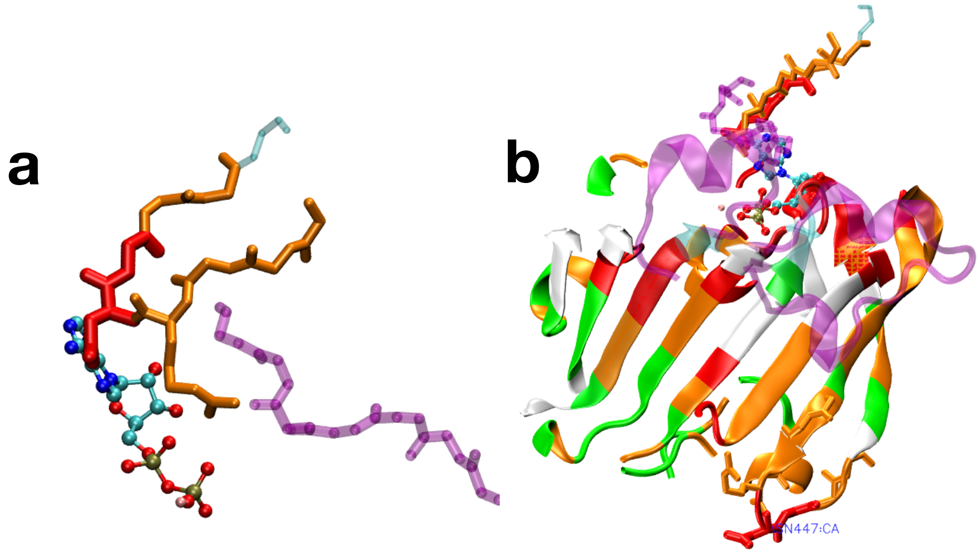


**Supplementary Figure 15:** *Site-specifically identified conformational changes in* **a** the R-finger and **b** central half-beta barrel upon ADP:$AlF_4^-$ binding.

# Supplementary Tables

**Supplementary Table 1:** Assignment statistics for all studied protein-complexes. The percentage of assigned CTD (residues 177-488) Cα resonances is given. Note that a significant fraction of the CTD is entirely dynamic and was therefore detected in previous work in $^{1}H,^{13}C$ INEPT studies, but remained completely unassigned up to now[5].

| **Protein-complex** | **Assigned Cα resonances of CTD/ %** |
|---|---|
| apo | 31 |
| AMP-PCP | 25 |
| AMP-PCP:DNA | 37 |
| ADP:$AlF_4^-$ | 33 |
| ADP:$AlF_4^-$:ssDNA | 50 |
| ADP | 29 |
| ADP:DNA | 39 |

**Supplementary Table 2:** New appearing resonances during the ATP hydrolysis cycle which are marked in pink in Figure 2 of the main text.

| *apo*→AMP-PCP | AMP-PCP→ADP:$AlF_4^-$ | ADP: $AlF_4^-$→APD | ADP→ *apo* |
|---|---|---|---|
| 214, 387 | 200, 206, 207, 209, 211, 213, 230, 251, 271, 312, 314, 353, 382, 383, 390, 435, 447, 448, 458, 459, 461, 462 | none | 235, 251, 271, 313, 353, 356, 447 |

**Supplementary Table 3:** Disappearing resonances during the ATP hydrolysis cycle.

| *apo*→AMP-PCP | AMP-PCP→ADP:$AlF_4^-$ | ADP: $AlF_4^-$→APD | ADP→ *apo* |
|---|---|---|---|
| 207, 251, 271, 312, 313, 314, 353, 354, 356, 447 | none | 200, 206, 209, 211, 213, 235, 271, 382, 383, 435, 457, 461, 462 | 181, 204, 207, 214, 387, 390 |

**Supplementary Table 4**: Details of fluorescence anisotropy fits.

| | ATP | ADP | AMP-PCP | ADP:AlF$_4^-$ |
|---|---|---|---|---|
| Best-fit values | | | | |
| $B_{max}$ | 249.9 | 250 | 244.5 | 259.9 |
| $h$ | 0.6509 | 0.5361 | 0.7349 | 1.160 |
| $K_d$ | 14.42 | 5077 | 47.43 | 0.8349 |
| Std. Error | | | | |
| $B_{max}$ | 6.367 | N/A | 2.581 | 1.989 |
| $h$ | 0.05069 | 0.03174 | 0.02598 | 0.06161 |
| $K_d$ | 2.204 | 412.1 | 2.648 | 0.03348 |
| 95% Confidence Intervals | | | | |
| $B_{max}$ | 236.6 to 263.1 | N/A | 239.1 to 249.9 | 255.7 to 264.1 |
| $h$ | 0.5452 to 0.7567 | 0.4697 to 0.6026 | 0.6803 to 0.7895 | 1.031 to 1.290 |
| $K_d$ | 9.820 to 19.01 | 4214 to 5939 | 41.86 to 52.99 | 0.7646 to 0.9052 |
| Goodness of Fit | | | | |
| Degrees of Freedom | 20 | 19 | 18 | 18 |
| R square | 0.9920 | 0.9886 | 0.9984 | 0.9984 |
| Absolute Sum of Squares | 1801 | 586.7 | 249.2 | 434.1 |
| Sy.x | 9.489 | 5.557 | 3.721 | 4.911 |
| Constraints | None | Bmax=250 | None | None |

**Supplementary Table 5:** Overview of $^{31}$P NMR parameters for the various complexes of *Hp*DnaB.

| | **AMP-PCP** | **AMP-PCP:DNA** | **ADP:AlF$_4^-$** | **ADP:AlF$_4^-$:DNA** | **ADP** | **ATP** | **ADP:DNA** | **ATP:DNA** |
|---|---|---|---|---|---|---|---|---|
| **$\Delta\delta(^{31}P^{\alpha}-^{31}P^{\beta})$/ ppm** | 20.2 | 27.8 | 1.3 | 1.2 | 7.2 | 7.2 | 3.4 | 3.4 |
| **$\delta(^{31}P$, ssDNA)/ ppm** | - | 0.2; -1.4 | - | 0.5; -1.1 | - | - | 0.3; -1.2 | 0.5; -1.1 |

**Supplementary Table 6**: Appearing and disappearing resonances during DNA translocation highlighted in Figure 4 and Supplementary Figure 12.

| | AMP-PCP:ssDNA→ADP:AlF$_4^-$:ssDNA | ADP:AlF$_4^-$:ssDNA → ADP:ssDNA | ADP:ssDNA → AMP-PCP:ssDNA |
|---|---|---|---|
| Appearing | 204, 206, 207, 209, 271, 381 | none | 211 |
| Disappearing | none | 206, 211, 381 | 204, 207, 209, 271 |

## Supplementary References


1. Itsathitphaisarn, O., Wing, Richard A., Eliason, William K., Wang, J. & Steitz, Thomas A. The Hexameric Helicase DnaB Adopts a Nonplanar Conformation during Translocation. *Cell* **151**, 267-277 (2012).
2. Bazin, A., Cherrier, M.V., Gutsche, I., Timmins, J. & Terradot, L. Structure and primase-mediated activation of a bacterial dodecameric replicative helicase. *Nucleic Acids Res.* **43**, 8564-8576 (2015).
3. Strycharska, M.S. et al. Nucleotide and partner-protein control of bacterial replicative helicase structure and function. *Molecular cell* **52**, 844-854 (2013).
4. Wiegand, T. et al. Variability and conservation of structural domains in divide-and-conquer approaches. *J. Biomol. NMR* **65**, 79-86 (2016).
5. Wiegand, T., Cadalbert, R., von Schroetter, C., Allain, F.H.-T. & Meier, B.H. Segmental isotope labelling and solid-state NMR of a 12 × 59 kDa motor protein: identification of structural variability. *J. Biomol. NMR* **71**, 237-245 (2018).
6. Wiegand, T. et al. Monitoring ssDNA Binding to the DnaB Helicase from Helicobacter pylori by Solid-State NMR Spectroscopy. *Angew. Chem. Int. Ed.* **55**, 14164-14168 (2016).
