# Supplementary material for: The conformational changes coupling ATP hydrolysis and translocation in a bacterial DnaB helicase
Source: Nat Commun. 2019 Jan 3;10:31. doi: 10.1038/s41467-018-07968-3 (PMC6318325; doi:10.1038/s41467-018-07968-3)
Supplement: Supplementary file 3 — Reporting Summary [file 41467_2018_7968_MOESM3_ESM.pdf]

nature research

Corresponding author(s): A. Böckmann
B. H. Meier

Reporting Summary

Nature Research wishes to improve the reproducibility of the work that we publish. This form provides structure for consistency and transparency in reporting. For further information on Nature Research policies, see Authors & Referees and the Editorial Policy Checklist.

Statistical parameters

When statistical analyses are reported, confirm that the following items are present in the relevant location (e.g. figure legend, table legend, main text, or Methods section).

n/a	Confirmed	
☐	☒	The exact sample size (n) for each experimental group/condition, given as a discrete number and unit of measurement
☐	☒	An indication of whether measurements were taken from distinct samples or whether the same sample was measured repeatedly
☒	☐	The statistical test(s) used AND whether they are one- or two-sided<br>*Only common tests should be described solely by name; describe more complex techniques in the Methods section.*
☒	☐	A description of all covariates tested
☒	☐	A description of any assumptions or corrections, such as tests of normality and adjustment for multiple comparisons
☐	☒	A full description of the statistics including central tendency (e.g. means) or other basic estimates (e.g. regression coefficient) AND variation (e.g. standard deviation) or associated estimates of uncertainty (e.g. confidence intervals)
☒	☐	For null hypothesis testing, the test statistic (e.g. F, t, r) with confidence intervals, effect sizes, degrees of freedom and P value noted<br>*Give P values as exact values whenever suitable.*
☒	☐	For Bayesian analysis, information on the choice of priors and Markov chain Monte Carlo settings
☒	☐	For hierarchical and complex designs, identification of the appropriate level for tests and full reporting of outcomes
☒	☐	Estimates of effect sizes (e.g. Cohen's d, Pearson's r), indicating how they were calculated
☐	☒	Clearly defined error bars<br>*State explicitly what error bars represent (e.g. SD, SE, CI)*

Our web collection on statistics for biologists may be useful.

Software and code

Policy information about availability of computer code

Data collection	Bruker Topspin (https://www.bruker.com/products/mr/nmr/nmr-software/software/topspin/overview.html?gclid=EAIaIQobChMImvnRisrR3glVT_lRCh0G8gRzEAAYASAAEgLzcPD_BwE)
Data analysis	CCPN analysis (https://www.ccpn.ac.uk/v2-software/software/analysis), OriginPro (https://www.originlab.com/Origin), Microsoft Excel, VMD (https://www.ks.uiuc.edu/Research/vmd/), GraphPad Prism6 (https://www.graphpad.com/scientific-software/prism/)

For manuscripts utilizing custom algorithms or software that are central to the research but not yet described in published literature, software must be made available to editors/reviewers upon request. We strongly encourage code deposition in a community repository (e.g. GitHub). See the Nature Research guidelines for submitting code & software for further information.

Data

Policy information about availability of data

All manuscripts must include a data availability statement. This statement should provide the following information, where applicable:

- Accession codes, unique identifiers, or web links for publicly available datasets
- A list of figures that have associated raw data
- A description of any restrictions on data availability

Data supporting the findings of this manuscript are available from the corresponding author upon reasonable request. The following PDB structures were used in

this study: 4ZC0 (http://dx.doi.org/10.2210/pdb4ZC0/pdb) and 4NMN (http://dx.doi.org/10.2210/pdb4NMN/pdb).
The source data underlying Figs 2, 3 and 4 and Supplementary Figs S7 and S12 as well as the chemical-shift values for apo DnaB and DnaB:ADP:AlF4-:ssDNA and all experimental NMR parameters are provided as a Source Data file.

Field-specific reporting

Please select the best fit for your research. If you are not sure, read the appropriate sections before making your selection.

☒ Life sciences ☐ Behavioural & social sciences ☐ Ecological, evolutionary & environmental sciences

For a reference copy of the document with all sections, see nature.com/authors/policies/ReportingSummary-flat.pdf

Life sciences study design

All studies must disclose on these points even when the disclosure is negative.

Sample size	No sample-size calculation was performed.
Data exclusions	No data were excluded.
Replication	1D and in most cases also 2D NMR experiments were repeated before measuring 3D spectra to check the sample stability. Most samples were at least prepared two times and the spectra were identical.
Randomization	Not relevant for NMR.
Blinding	Not relevant for NMR.

Reporting for specific materials, systems and methods

Materials & experimental systems

n/a	Involved in the study	
☒	☐	Unique biological materials
☒	☐	Antibodies
☒	☐	Eukaryotic cell lines
☒	☐	Palaeontology
☒	☐	Animals and other organisms
☒	☐	Human research participants

Methods

n/a	Involved in the study	
☒	☐	ChIP-seq
☒	☐	Flow cytometry
☒	☐	MRI-based neuroimaging
